# Aspirin plus clopidogrel versus aspirin alone for the prevention of coronary artery bypass graft occlusion: angiographic and clinical results of a randomized study

**DOI:** 10.1186/1749-8090-10-S1-A127

**Published:** 2015-12-16

**Authors:** Maria J Dalmau, Manuel Barreiro, Javier López-Rodríguez, Maria Bueno, Elena Arnáiz, Adolfo Arévalo, Ana Martín, Jose M González-Santos

**Affiliations:** 1 Department of Cardiac Surgery, Salamanca University Hospital, Salamanca, 37007, Spain; 2 Department of Cardiology, Salamanca University Hospital, Salamanca, 37007, Spain

## Background/Introduction

Prevalence of graft occlusion is high after coronary artery bypass grafting (CABG). Routine use of aspirin after CABG reduces graft failure and ischemic complications. The benefit of concomitant clopidogrel administration remains a controversial issue.

## Aims/Objectives

We sought to evaluate the impact of use aspirin plus clopidogrel versus aspirin alone on graft patency and adverse cardiovascular events 18 months after CABG.

## Method

In this single-centre prospective randomized study, 200 consecutive patients undergoing elective CABG were randomly assigned to two groups: 97 patients received aspirin 300 mg (A) and 103 patients received aspirin 100 mg plus clopidogrel 75 mg (AC). Antiplatelet protocol was initiated immediately after surgery and continued daily for one year. Graft patency was evaluated by multislice computed tomography angiography (MSCT) 18 months after surgery. The occurrence of major cardiac or cerebrovascular adverse events (MACCE) and bleeding complications during follow-up were assessed.

## Results

Preoperative patient characteristics were similar in both groups. MSCT graft patency was assessed in 194 patients (97%). A total of 542 grafts and 680 distal anastomoses were analyzed. Overall graft patency was 83.1% in group A and 89.4% in the AC group (p = 0.04). Patency rates in the A group versus AC group were similar: Left internal mammary artery (95.9% vs. 95.1%, p = 0.85), right internal mammary artery (97.8% vs. 91.2%, p = 0.37), radial artery (74.2% vs. 92.6%, p = 0.06), saphenous vein graft (78.6% vs. 83.5%, p = 0.45). Clinical follow-up was complete in 200 patients. The incidence of MACCEs was similar in both groups (A 5.2% vs. AC 9.7%, p = 0.22). The need for percutaneous coronary re-intervention showed no statistically significant differences (A 3.1% vs. AC 4.9%, p = 0.78).

## Discussion/Conclusion

Compared with aspirin monotherapy, the combination of aspirin plus clopidogrel following CABG did not significantly increase graft patency or reduce the incidence of adverse cardiac events.

